# Evaluation of Anti-proliferative Effects of *Barringtonia racemosa* and Gallic Acid on Caco-2 Cells

**DOI:** 10.1038/s41598-020-66913-x

**Published:** 2020-06-19

**Authors:** Ivan Y. M. Ho, Azlina Abdul Aziz, Sarni Mat Junit

**Affiliations:** Department of Molecular Medicine, Faculty of Medicine, University of Malaya, 50603 Kuala Lumpur, Malaysia

**Keywords:** Transcription, Cancer, Biochemistry, Drug discovery, Molecular biology

## Abstract

*Barringtonia racemosa* leaf water extract (BLE) had been shown to have high gallic acid (GA) content and BLE has been postulated to have anti-proliferative effects towards colorectal cancer. This study aims to further investigate the mechanism underlying the anti-proliferative effect of BLE in Caco-2 cells and to determine if GA is responsible for the observed effects. Both BLE and GA inhibited Caco-2 cells in a dose-dependent manner. Cells exposed to IC_50_ concentration of BLE and GA showed reduced antioxidant activities. GA-treated Caco-2 cells experienced higher oxidative stress compared to cells treated with BLE. Both BLE and GA significantly up-regulated the expression of *SLC2A1*. BLE but not GA, significantly down-regulated the expression of *ADH4*. Meanwhile, GA but not BLE, significantly up-regulated *AKRIB10* and *GLO1* but significantly down-regulated *HAGH*. Alterations in gene expression were coupled with changes in extracellular glucose and pyruvate levels. While BLE decreased intracellular pyruvate, GA did the opposite. Both intracellular and extracellular D-lactate were not affected by either BLE or GA. GA showed more pronounced effects on apoptosis while BLE irreversibly reduced cell percentage in the G0/G1 phase. In conclusion, this study demonstrates the multiple-actions of BLE against Caco-2 cells, potentially involving various polyphenolic compounds, including GA.

## Introduction

*Barringtonia racemosa* (L.) Spreng is a medicinal plant belonging to the Lecythideceae family that is commonly found throughout Eastern Africa, Polynesia, Africa and Asia including Malaysia^1^. In Malaysia, the shoot and young leaves of *B. racemosa* are usually consumed raw as a salad with various condiments, and different parts of the plant are used in traditional medicine^2^.

Studies on various parts of *B. racemosa* have demonstrated its biological activities that includes antibacterial^3^, antifungal^4^, antioxidant^5^, anti-inflammatory^6^ and anti-cancer^7–9^. Initial analysis performed by our group indicated that the leaf water extract of *B. racemosa* (BLE) had the highest polyphenolic and ascorbic acid content as well as antioxidant activities amongst various extracts prepared in solvents of different polarities, including water, ethanol, ethyl acetate and hexane^10^. Ultra-high performance liquid chromatography (UHPLC) analyses of the BLE revealed the presence of gallic acid (GA), protocathechuic acid, ellagic acid, quercetin, kaempferol and rutin^10,11^.

The anti-proliferative activities of *B. racemosa* leaf extract was reported against cervical cancer cell line, HeLa^7^, tumour in mice challenged with Dalton’s Lymphoma Ascitic cells^9^ and leukemic cell lines, MOLT-3 and REH^8^. A recent study conducted by our group found that BLE had a direct effect on the regulation of gene expression in HepG2 cells^12^. Further *in-silico* analysis using Ingenuity Pathway Analysis (IPA) software revealed that the effect of BLE was associated to “cancer, cell death and survival and cell movement” and “cell cycle, connective tissue development and function, cellular development”, with the expression of several genes associated to colorectal cancer being significantly altered^12^. The leading pathway predicted to be affected by BLE was identified as “Methylglyoxal degradation III”^12^.

Methylglyoxal (MG) is a highly reactive α-oxoaldehyde that is produced as a by-product of glycolysis. The anti-cancer effects of MG against malignant cells in animals have also been previously reviewed^13^. A recent study also showed that the combination of MG and silencing of glyoxalase I (GLO1), the enzyme responsible for MG detoxification, can inhibit *in-vitro* SW620 colon cancer as well as *in-vivo* SW620 colon cancer xenograft model in mice^14^. The concentration of MG in cancer cells is hypothesized to be higher than in normal cells due to their high glycolytic rates^15^. However, it was found that cancer cells have reduced MG and elevated lactic acid concentration^16^. Several cancer types, including breast^17^, melanoma^18^ and colon cancers^19^, were reported to have overexpression of GLO1, suggesting that cancer cells have higher rates of MG degradation. Moreover, a recent report revealed the hormetic effects of MG, whereby MG exhibited low-dose stimulation and high-dose inhibition of tumor growth^20^. As such, it is possible to magnify the anti-cancer effects of MG by inhibiting MG degradation mechanisms, including MG degradation III targeted by BLE.

Thus, in the present study we applied biochemical and molecular approaches to investigate the effects of BLE on the antioxidant status and anti-proliferation of colorectal cancer cells Caco-2. We also investigated the potential role of the glycolytic pathway as one of the possible mechanisms responsible for the anti-proliferative effects of BLE. In addition, the effects of BLE were also compared with gallic acid, GA. GA was chosen as the comparative control as it was previously identified as the most abundant polyphenolic compound in BLE (Kong, Mat-Junit, Ismail, Aminudin & Abdul-Aziz, 2014)^11^ and it has high cytotoxicity against Caco-2 cells (Forester & Waterhouse, 2010)^21^. The result in this study may elucidate more information on the action mechanism of BLE against colorectal cancer cells.

## Materials and methods

### Cell culture

Human colon adenocarcinoma Caco-2 cell line was obtained from American Type Culture Collection, ATCC (Manassas, VA). The cells were cultured in complete Minimum Essential Media (MEM) with Earle’s salt (Nacalai Tesque, Kyoto, Japan) supplemented with 10% foetal bovine serum (FBS) (Sigma, St. Louis, MO) and 100 units/ml penicillin-streptomycin mixture (Nacalai Tesque, Kyoto, Japan) for complete growth. The cell cultures were maintained in humidified atmosphere at 37 °C and 5% CO_2_.

### Sample preparation and extraction

*Barringtonia racemosa* shoots were collected in Selangor, Malaysia, and a sample was deposited in the Herbarium of Rimba Ilmu, University of Malaya (Voucher no. KLU 48175). The sample preparation and extraction were performed according to Kong *et al*.^10^. Briefly, 224.5 g of the fresh leaves of *B. racemosa* were weighed and cleaned with distilled water. The leaves were then lyophilised using a freeze drier (Heto Lab Equipment Corp., Denmark), ground to powder and extracted with distilled water (2 g leaf powder/40 ml water) for 24 hours. The resulting extract was lyophilised and stored at −20 °C for further analysis. BLE was prepared by dissolving the dried BLE powder in complete MEM, to the desired concentration.

### Cytotoxicity effects of BLE and gallic acid

Caco-2 cells were first seeded in 96 well plates at a concentration of 5 × 10^3^ cells per well and grown in 200 µl complete MEM for 48 hours. Subsequently, the complete MEM was replaced with 200 μl complete MEM containing BLE with concentrations between 0 and 500 μg/ml. The cells were then incubated for another 48 hours prior to cell viability analysis using 3-(4,5-dimethylthiazol-2-yl)-2,5-diphenyltetrazolium bromide (MTT) assay. Briefly, complete MEM was replaced with 200 μl serum-free MEM together with 20 μl 5 mg/ml MTT and the cells were further incubated for 4 hours. Subsequently, the serum-free MEM and MTT were removed, and the formazan crystals formed were solubilised in 200 μl of dimethyl sulfoxide (DMSO). The purple solution was quantitated by measuring the absorbance at 570 nm (Tecan Infinite M1000 Pro, Switzerland). A dose-response curve was plotted based on the values of percentage of cell growth inhibition caused by BLE. The cell cytotoxicity of BLE was expressed as the effective concentration in μg/ml that corresponded to 20% (IC_20_) and 50% (IC_50_) cell growth inhibition. Complete MEM was used as negative control while complete MEM containing GA (3.1 to 500 µg/ml) (Sigma, St. Louis, MO) was used as comparative controls.

### Cell treatment with BLE and GA at IC_20_ and IC_50_ concentrations

Caco-2 cells used for the treatment was prepared by stabilizing 3.8 × 10^5^ cells in 25 cm^2^ culture flask containing complete MEM under humidified atmosphere (37 °C, 5% CO_2_) for 48 hours. The IC_20_ (69.1 µg/ml) and IC_50_ (325.5 µg/ml) concentrations of BLE were used for the cell treatment. Complete MEM was used as a negative control and complete MEM containing GA at concentrations that corresponds to the IC_20_ (3.7 µg/ml) and IC_50_ (10.6 µg/ml) were used as comparative controls. The cells were further incubated for 48 hours prior to analyses. Cells used for analyses were either collected as whole cells or sonicated to obtain the cell lysates.

Coomassie Brilliant Blue (CBB) solution (5×) (Nacalai Tesque, Kyoto, Japan) was diluted at a ratio of 1 CBB solution: 4 deionised water. Briefly, 10 µl of diluted CBB solution was mixed with 200 μl cell lysate in a 96-well plate and incubated for 10 minutes. After the incubation period, absorbance was read at 595 nm and the concentration of protein was determined by comparing the absorbance value against a standard curve plotted using bovine serum albumin (BSA) at concentrations between 0 and 1000 μg/ml.

### Cellular antioxidant status and parameters of oxidative stress

Ferric reducing antioxidant power (FRAP) assay was performed according to the method of ^22^ with slight modifications. Briefly, 50 µl of cell lysate was mixed with 175 µl of FRAP reagent in a 96-well plate, incubated in the dark at 37 °C for 30 minutes and quantitated by measuring the absorbance at 595 nm. A standard calibration curve was plotted using the absorbance value of iron (II) sulfate solution with concentrations between 0 to 1000 μmol/ml. The ferric reducing activity of BLE was expressed as μmol of Fe^2+^/g dried weight of BLE.

2,2′-azino-bis (3-ethylbenzothiazoline-6-sulphonic acid) (ABTS) radical scavenging assay was performed according to^23^ with slight modifications. Two microliters of cell lysate were mixed with 200 μl of ABTS radical solution in a 96-well plate, incubated in the dark for 6 minutes and quantitated by measuring the absorbance at 734 nm. ABTS radical scavenging ability of cell lysates was expressed as percentage inhibition of the ABTS radicals.

Reactive oxygen species (ROS) production in the cells was measured according to^24^ with slight modifications. Caco-2 cells treated with BLE in 25 cm^2^ culture flasks were collected and diluted with phosphate-buffered saline (PBS) to 2 × 10^5^ cells/ml. Fifty microliters of Caco-2 cell suspension was mixed with 50 μl of 20 μM dichloro-dihydro-fluorescein diacetate (DCFH-DA) solution in a 96-well plate and incubated in the dark at 37 °C for 45 minutes. The plate was read at excitation of 485 nm and emission of 520 nm using Infinite M1000 Pro multimode reader and its corresponding i-control software (Tecan, Männedorf, Switzerland).

Lipid peroxidation in the cells was determined by measuring malondialdehyde (MDA) produced in the presence of thiobarbituric acid (TBA) according to^25^. Briefly, 500 μl TBA reagent was mixed with 100 μl cell lysate, heated at 90 °C for 20 minutes and cooled on ice for 5 minutes. The resulting mixture was centrifuged at 3000 × *g* for 10 minutes and 100 μl of the supernatant was transferred to a 96-well plate. MDA was quantitated by measuring the absorbance at 535 nm. MDA standard curve was prepared using 1,1,3,3-tetraethoxy propane (TEP) with concentrations between 0 to 4 μM.

Activities of the antioxidant enzymes were measured using commercially available assay kits from Cayman Chemical Company (Ann Arbor, MI). The antioxidant enzymes tested were catalase (CAT), glutathione peroxidase (GPx) and superoxide dismutase (SOD). Cellular lysates were prepared by collecting the treated cells using a rubber policeman, followed by sonication at high speed (PowerSonic 405 Ultrasonicator, Korea) for 5 minutes. Analyses of antioxidant enzyme activities of the cell lysates were performed following the manufacturer’s guide.

### RNA isolation and quantitative reverse transcription-PCR (qRT-PCR)

RNA extraction was performed according to the manufacturer’s protocol (Total RNA Mini Kit, Geneaid Biotech, New Taipei City, Taiwan). The RNA extracted was then reverse-transcribed using ReverTra Ace qPCR RT Master Mix (Toyobo, Osaka, Japan). Fifty nanograms of the resulting cDNA were mixed with primers and Thunderbird SYBR qPCR Mix (Toyobo). StepOnePlus System and the corresponding StepOne Software v2.3 (Applied Biosystems, Carlsbad, CA) were used for qRT-PCR. *GAPDH* and *RPLP0* were used for relative gene expression normalisation. Primers were synthesised by Integrated DNA Technologies (IDT) (Coralville, IA) and Macrogen (Seoul, South Korea). The primer sequences are detailed in Supplementary Table 1. The expression of genes, in fold change, at equal or greater than 1.5 folds at p < 0.05 is considered as significant.

### Quantification of cellular metabolites

The metabolites quantified were glucose, D-lactate and pyruvate. Glucose (HK) assay kit was purchased from Sigma (St. Louis, MO) while D-lactate and pyruvate assay kits were sourced from Cayman Chemical Company (Ann Arbor, MI). Intracellular glucose concentration was determined by measuring the glucose concentration of the cellular lysate while extracellular glucose concentration was determined by measuring glucose concentration of the culture media after treatment. Intracellular D-lactate, extracellular D-lactate, intracellular pyruvate and extracellular pyruvate concentrations were measured using similar protocol as glucose measurement, utilising the D-lactate and pyruvate assay kits.

### Cell cycle and apoptosis

Flow-cytometry analyses were performed using the Muse Cell Analyzer system and Muse 1.5 Analysis software (Merck, Darmstadt, Germany). For cell cycle analysis, the cells collected were first fixed in 70% ethanol for 12 hours, diluted to 3 × 10^5^ cells/ml with Muse Cell Cycle Reagent (Merck) and incubated in the dark for 30 minutes prior to analysis. The number of events for analysis was set at 5000.

The cell cycle analyses were repeated but following a slightly different protocol whereby the media for cells that have been treated with BLE and GA at IC_20_ and IC_50_ for 48 hours, were replaced with fresh MEM, followed by a further 48-hours incubation (37 °C and 5% CO_2_)^26^. Flow cytometry analyses were then performed as above.

The results for cell cycle analysis were expressed as DNA content profiles. For apoptosis analysis, Caco-2 cells were collected and resuspended in complete MEM at a concentration of 6 × 10^5^ cells/ml. Two hundred microlitres of cell suspension was mixed with 200 μl of Muse Annexin V and Dead Cell Reagent (Merck) and incubated in the dark for 20 minutes prior to analysis. The number of events for analysis was set at 5000. The results for apoptosis analysis were expressed as apoptotic profiles.

### Statistical analysis

Results are presented as mean of percentage relative to negative control with standard error of the mean (SEM) of three independent biological replicates. All statistical analyses were performed using SPPS statistical software version 25.0 (SPSS Inc., Chicago, IL). One-way analysis of variance (ANOVA) was used to compare the means between groups with level of significance set at p < 0.05. Significant differences detected with ANOVA were further analysed using Dunnett t-test with negative control as the reference group.

## Results

### Cytotoxic effects of BLE and GA on Caco-2 cells

**Figure 1:**
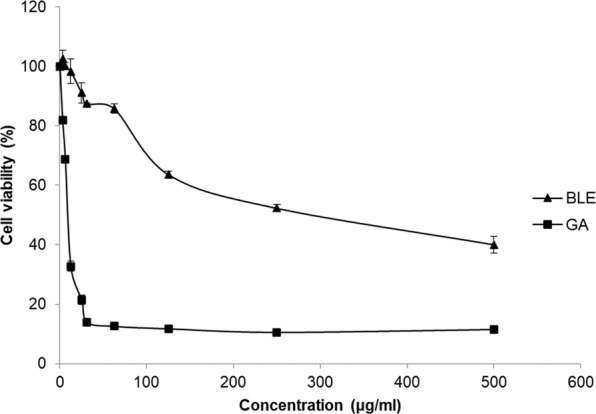
Cytotoxicity of BLE and GA on Caco-2 cell line evaluated by MTT assay after 48 hours of treatment. Caco-2 cells grown in MEM only, was used as a negative control. Data are presented as mean percentage cell viability ± SD of three independent biological replicates. MEM = Minimum essential media; BLE = *B. racemosa* leaf water extract; GA = gallic acid; and MTT = 3-(4,5-dimethylthiazol-2-yl)-2,5-diphenyltetrazolium bromide.

Caco-2 cells were treated with increasing doses (3.1 to 500 μg/ml) of BLE and GA for 48 hours and their viability was determined using MTT assays. Figure 1 shows the cell viability curve, expressed as percentage viability. BLE IC_20_ and BLE IC_50_ were found to be at 69.1 ± 3.2 μg/ml and 325.5 ± 12.8 μg/ml respectively while GA IC_20_ and GA IC_50_ were found to be at 3.7 ± 0.1 μg/ml and 10.6 ± 0.6 μg/ml respectively. BLE IC_20_, BLE IC_50_, GA IC_20_ and GA IC_50_ were selected for further analyses.

### Cellular antioxidant status and parameters of oxidative stress of Caco-2 cells treated with BLE, GA for 48 hours

**Figure 2:**
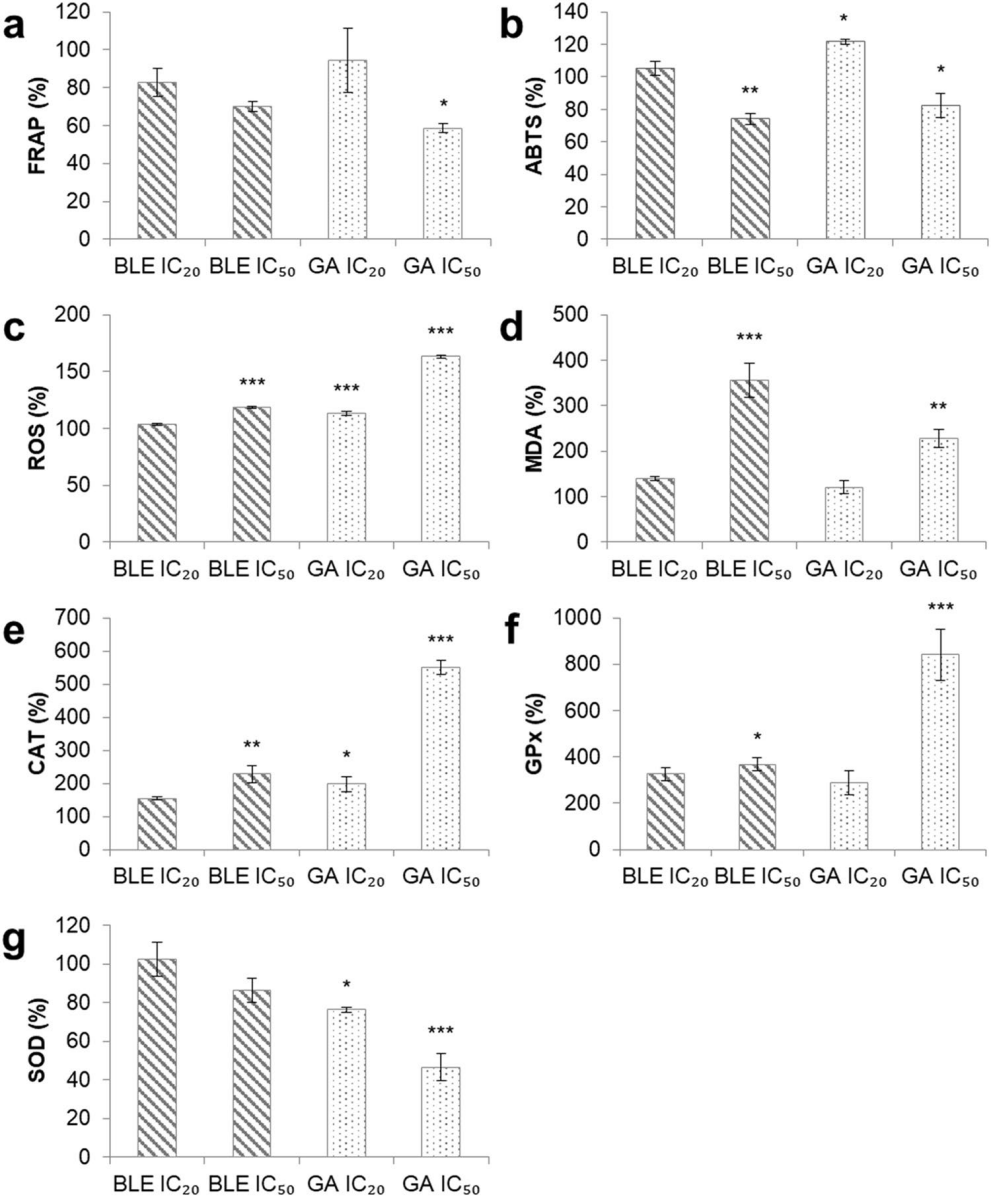
Cellular antioxidant status and parameters of oxidative stress of Caco-2 cells after treatment with BLE and GA for 48 hours. (**a**) FRAP of cell lysates (**b**) ABTS scavenging ability of cell lysates (**c**) ROS production of cells (**d**) MDA formation of cell lysates (**e**) Catalase activity of cells (**f**) Glutathione peroxidase activity of cells (**g**) Superoxide dismutase activity of cells. Caco-2 cells grown in MEM only, was used as the negative control. Data are presented as mean percentage change ± SEM of three independent biological replicates. *p < 0.05, **p < 0.01 and ***p < 0.001. MEM = Minimum essential media; BLE = *B. racemosa* leaf water extract; GA = gallic acid; FRAP = ferric reducing antioxidant power; ABTS = 2,2′-azino-bis(3-ethylbenzothiazoline-6-sulphonic acid); ROS = reactive oxygen species; and MDA = malondialdehyde.

Cellular antioxidant status of Caco-2 cells were assessed using FRAP and ABTS assays. BLE at IC_50_ showed both reduced ferric reducing (Fig. 2a) and ABTS radical scavenging (Fig. 2b) activities compared to the negative control, although this was only significantly different in the latter. On the other hand, GA at IC_20_ caused increased ABTS scavenging activity and no change in ferric reducing activity compared to the negative control. However, at the IC_50_ concentration, GA showed both reduced ferric reducing and ABTS radical scavenging activities (Fig. 2a,b)

ROS was assessed by measuring DCF fluorescence in Caco-2 cells in the presence of intracellular ROS. BLE at IC_50_ significantly induced ROS formation in Caco-2 cells by 119% while GA at IC_20_ and IC_50_ significantly induced ROS formation by 113% and 163%, respectively, relative to negative control (Fig. 2c).

Lipid peroxidation of Caco-2 cells was determined by measuring MDA formed in the presence of TBA. At IC_50_, the effect of BLE on lipid peroxidation of Caco-2 cells was greater than that of GA. Caco-2 cells treated with BLE IC_50_ had 356% higher MDA than the negative control while cells treated with GA IC_50_ had 228% higher MDA than the negative control (Fig. 2d).

Activities of the antioxidant enzymes CAT, GPx and SOD in Caco-2 cells were measured using conventional kits. BLE at IC_50_ significantly induced the activity of CAT and GPx by 228% (Fig. 2e) and 369% (Fig. 2f), respectively while the activity of SOD remained unchanged (Fig. 2g). GA at IC_20_ significantly induced the activity of CAT by 198% and reduced the activity of SOD by 76%. GA at IC_50_ showed the most drastic changes in CAT and GPx whereby their activities were induced by 551% and 841%, respectively while the activity of SOD was reduced by 47%, relative to negative control.

### Alteration of expression of genes associated with MG degradation and glycolytic processes in response to BLE and GA in Caco-2 cells

**Figure 3:**
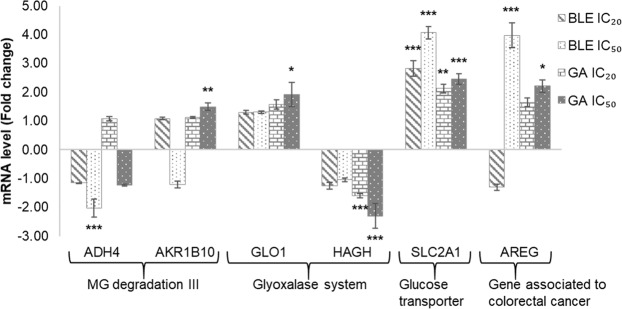
The expression of *ADH4*, *AKR1B10*, *AKR1C2*, *GLO1*, *HAGH*, *SLC2A1*, and *AREG* genes in Caco-2 cells treated with BLE and GA at IC_20_ and IC_50_ for 48 hrs. The expression of *GAPDH* and *RPLP0* genes were used as internal references. Caco-2 cells grown in MEM only, was used as the negative control. Data are presented as mean fold change ± SEM of three independent biological replicates. *p < 0.05, **p < 0.01 and ***p < 0.001. MEM = Minimum essential media; BLE = *B. racemosa* leaf water extract; and GA = gallic acid.

The expression of selected genes related to MG degradation III (*AKR1B10*, *AKR1C2* and *ADH4*), glyoxalase system (*GLO1* and *HAGH*), glucose transport (*SLC2A1* and *SLC5A1*) and colorectal cancer-associated genes (*AREG*, *CXCL8* and *CEACAM1*) were quantitated using qRT-PCR (Fig. 3). BLE IC_50_ significantly down-regulated the expression of *ADH4* by −2.03 folds (p < 0.001) but no significant changes in expression was detected for *AKR1B10* and *AKR1C2*. Meanwhile, treatment with BLE IC_20_ did not give any significant changes to the expression of genes associated with MG degradation III. In contrast, GA at IC_50_ significantly up-regulated the expression of *AKR1B10* by 1.5 fold (p < 0.01) but had no effects on the expression of *AKR1C2* and *ADH4*. Only treatment with GA, at both IC_20_ and IC_50_, significantly altered the expression of genes related to the glyoxalase system. *GLO1* expression was significantly up-regulated by GA IC_50_, by 1.92 fold, while the expression of *HAGH* was significantly down-regulated by GA at IC_20_ and IC_50_, by – 1.60 and − 2.31 fold respectively. All treatments altered the expression of at least one of the genes associated with glucose transport. *SLC5A1* gene was not significantly expressed in all cells, including the untreated control. The expression of *SLC2A1* was up-regulated by all treatments, with the greatest effect observed in cells treated with BLE IC_50_ followed by BLE IC_20_, GA IC_50_ and GA IC_20_ with a fold change difference of 4.07, 2.82, 2.46 and 2.13, respectively. For genes associated with colorectal cancer cells, only the expression of *AREG* was significantly up-regulated by BLE IC_50_ and GA IC_50_, by 3.97 and 2.21 folds respectively. The expression of *CXCL8* was not significantly altered while *CEACAM1* was not significantly expressed in all cells, including the untreated control.

### Evaluation of intracellular and extracellular levels of glucose, D-lactate and pyruvate in Caco-2 cells

**Figure 4:**
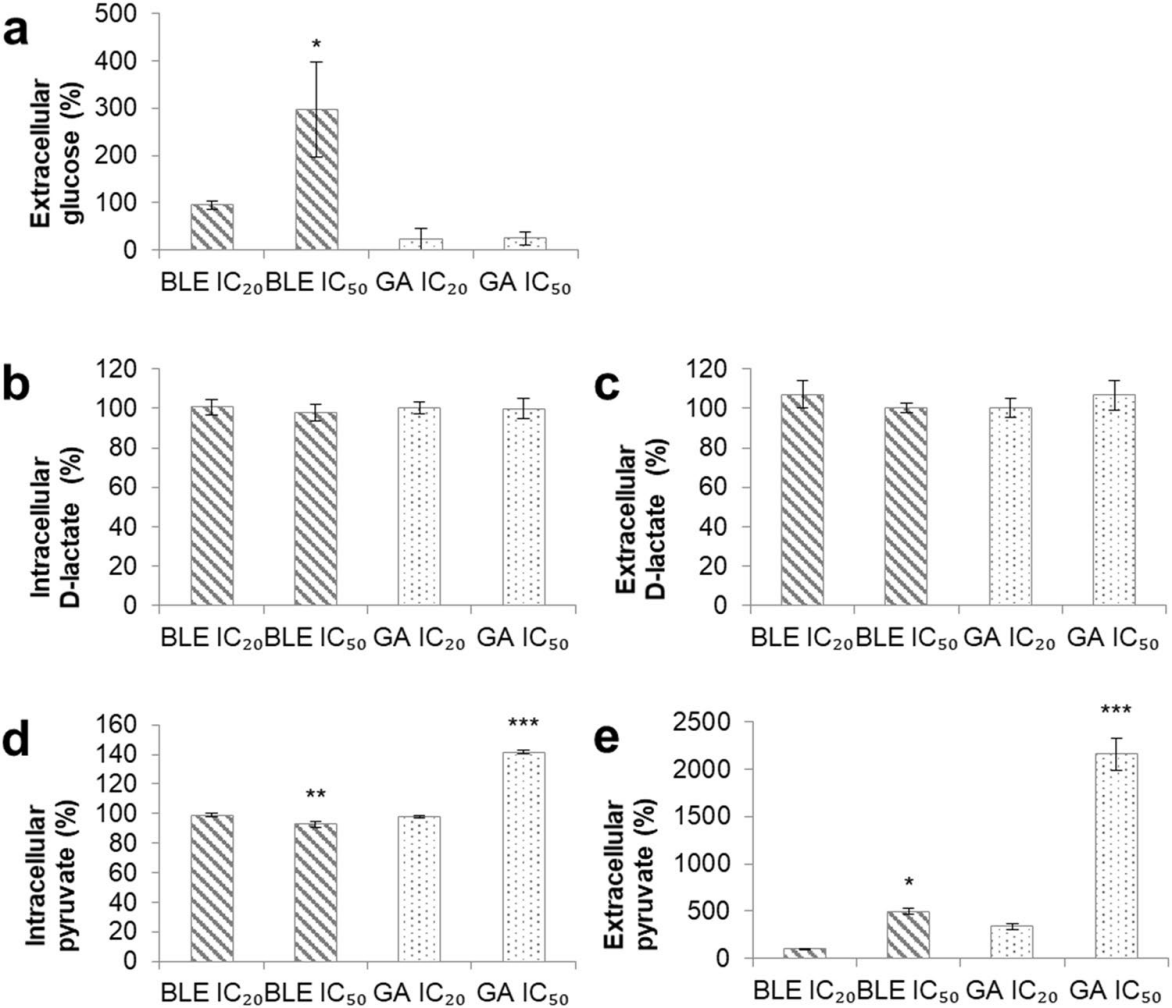
Changes in concentrations of glucose, D-lactate and pyruvate in Caco-2 cells treated with BLE and GA for 48 hours. (**a**) Extracellular glucose (**b**) Intracellular D-lactate (**c**) Extracellular D-lactate (**d**) Intracellular pyruvate (**e**) Extracellular pyruvate. Caco-2 cells grown in MEM only, was used as the negative control. Data are presented as mean percentage change ± SEM of three independent biological replicates. *p < 0.05, **p < 0.01 and ***p < 0.001. MEM = Minimum essential media; BLE = *B. racemosa* leaf water extract; and GA = gallic acid.

Treatment of Caco-2 cells with BLE IC_50_ caused a significant increase in extracellular glucose concentration, by 296%, compared to untreated cells while no significant changes were detected for cells treated with BLE IC_20_, GA IC_20_ and GA IC_50_ (Fig. 4a). Intracellular glucose concentration could not be determined as the concentration was lower than the detection limit of the glucose assay kit. There were no significant changes in both intracellular and extracellular D-lactate concentrations (Fig. 4b,c). However, the intracellular pyruvate concentration of Caco-2 cells treated with BLE IC_50_ was significantly reduced by 93% (Fig. 4d) while the extracellular pyruvate concentration was significantly increased by 497% (Fig. 4e). On the contrary, GA IC_50_ significantly induced both intracellular and extracellular pyruvate concentrations by 142% and 2157%, respectively.

### Cell cycle alteration of Caco-2 cells treated with BLE, GA

**Figure 5:**
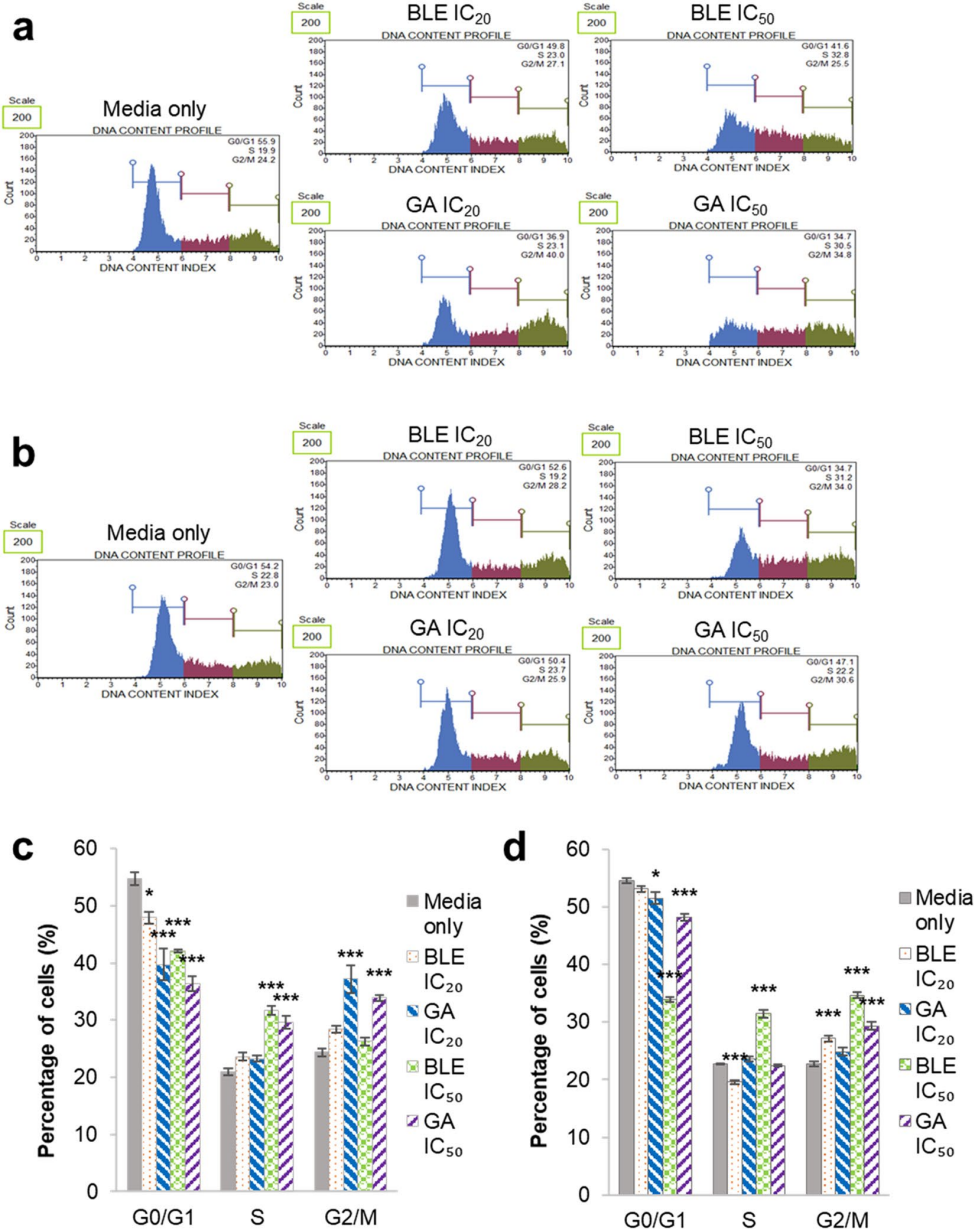
Cell cycle analyses of Caco-2 cells treated with BLE and GA. (**a**) DNA content profile of Caco-2 cells after 48 hours of treatment. (**b**) DNA content profile of Caco-2 cells after 48 hours of treatment followed by another 48 hours of incubation in fresh MEM (**c**) Cell distribution in different phases of cell cycle after 48 hours of treatment. (**d)** Cell distribution in different phases of cell cycle after 48 hours of treatment followed by another 48 hours of incubation in fresh MEM. Caco-2 cells grown in MEM only, was used as the negative control. Data are presented as mean percentage of cells ± SEM of three independent biological replicates. *p < 0.05, **p < 0.01 and ***p < 0.001. MEM = Minimum essential media; BLE = *B. racemosa* leaf water extract; and GA = gallic acid.

All treatments significantly reduced the percentage of cells in the G0/G1 phase (Fig. 5a). Meanwhile, only BLE IC_50_ and GA IC_50_ caused significant increase in the percentage of cells in the S phase (Fig. 5c) and only cells treated with GA show significantly higher percentage of cells in the G2/M phase. The ability of the cells to overcome the effects of BLE and GA treatments was analysed by substituting the treatment media with fresh MEM followed by a further 48 hours incubation. All treatments except BLE IC_50_ showed increased percentage of cells in G0/G1 phase after incubation in fresh media (Fig. 5d) compared to their respective treatments without media replacement (Fig. 5c). Interestingly, BLE at both concentrations resulted in significantly different percentage of cells in the S phase compared to the untreated cell with media replacement, with BLE IC_20_ showing a reduction and BLE IC_50_ showing an increment. Both concentrations of BLE caused significant increase in percentage of cells in the G2/M phase. The percentage of cells remained unchanged for both GA concentrations in the S phase while only GA IC_50_ resulted in significantly increased percentage of cells in the G2/M phase. The media replacement appears to reverse the effect of GA as the DNA content profile of cells treated with GA at both concentrations appeared to take the shape of that of the untreated cells (Fig. 5b).

### Apoptotic effects of BLE, GA on Caco-2 cells

**Figure 6:**
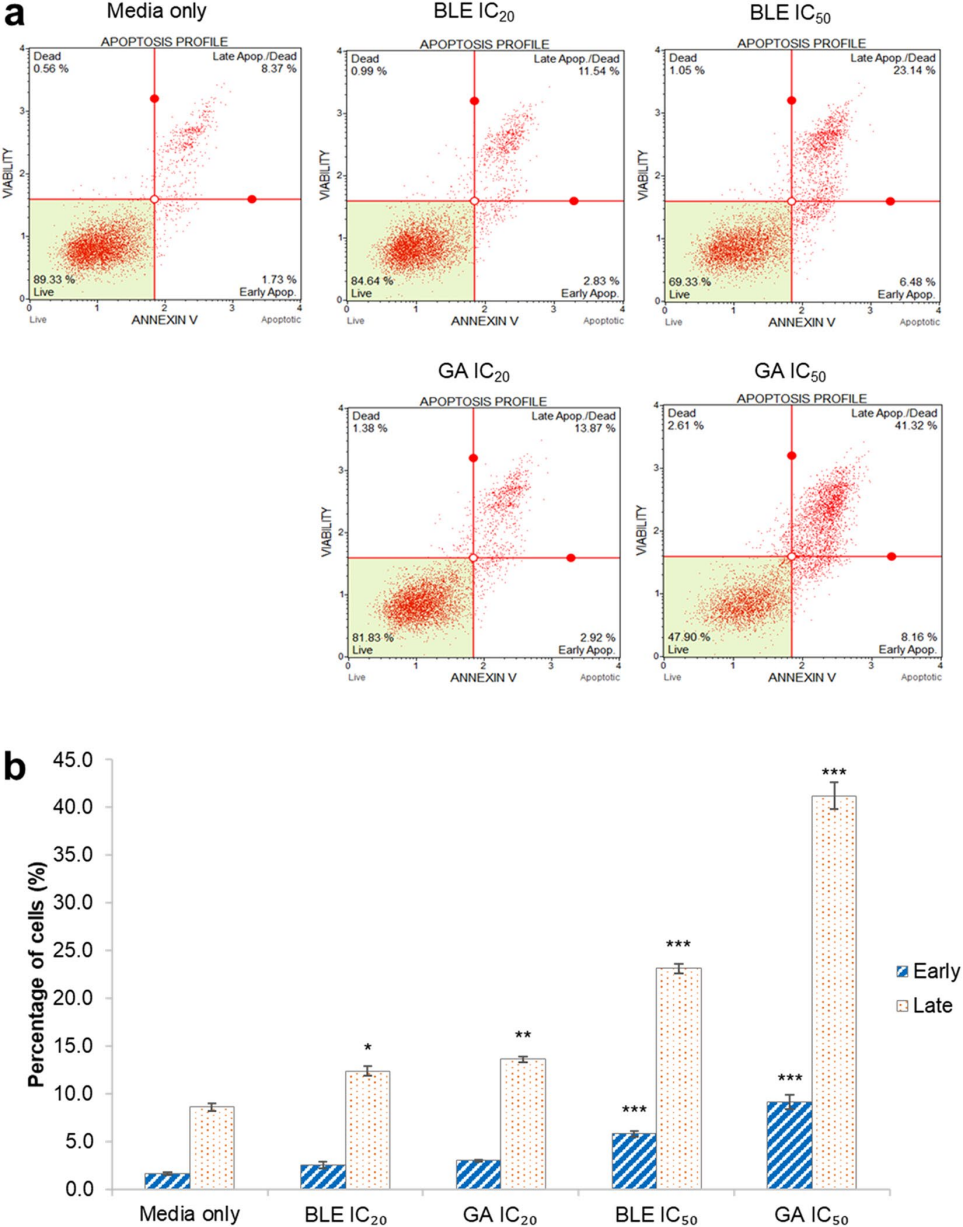
Apoptosis analysis of Caco-2 cells treated with BLE and GA. (**a**) Apoptosis profile of cells after 48 hours of treatment. (**b**) Percentage of cells undergoing early apoptosis and late apoptosis. Caco-2 cells grown in MEM only, was used as the negative control. Data are presented as mean percentage of cells ± SEM of three independent biological replicates. *p < 0.05, **p < 0.01 and ***p < 0.001. MEM = Minimum essential media; BLE = *B. racemosa* leaf water extract; and GA = gallic acid.

All treatments reduced the percentage of viable cells and induced apoptosis. In both BLE and GA-treated Caco-2 cells, the percentage of cells undergoing apoptosis increased with the concentration of treatment (Fig. 6a,b). However, only treatments with either BLE IC_50_ or GA IC_50_ caused significant increase in percentage of cells undergoing early apoptosis, by 5.8% and 9.2%, respectively. The apoptotic effect of GA is greater than that of BLE as shown in the percentage of cells undergoing apoptosis at both IC_20_ and IC_50_ concentrations.

## Discussion

Our group had previously reported the presence of GA, ellagic acid, protocatechuic acid, quercetin, rutin, and kaempferol in BLE^10,11^. Microarray and in-silico IPA analyses revealed the ability of BLE to alter the expression of genes in HepG2 cells including those associated to methylglyoxal (MG) degradation and glycolytic processes with colon cancer as a potential selective target^12^. GA, the most abundant polyphenolic compound in BLE, was selected as a comparative control for this study. UHPLC analyses on BLE sample used in this study confirmed the presence of GA, the polyphenolic compound that was previously identified by our group (Supplementary Fig. 1). The present study demonstrated the anti-proliferative effects of BLE against colorectal cancer cell model, Caco-2. The IC_50_ concentration for GA in this study was consistent with those reported in Caco-2^21^ and HepG2 cells^27^. Meanwhile, Kong *et al*.^27^ also reported that HepG2 cells treated with BLE at concentrations below 200 µg/ml resulted in more than 90% cell viability. The treatment of Caco-2 cells in this study with 31.25 µg/ml BLE resulted in cell viability below 90%. This suggests that BLE may have greater specificity towards Caco-2 cells than HepG2 cells.

Different research groups have reported the anticancer effects of various natural compounds. Ferhi *et al*.^28^ demonstrated that grape leaf water extract can influence the expression of apoptosis-related genes in HepG2 cells and breast cancer cell line, MCF-7 while Miceli *et al*.^29^ demonstrated that methanolic *Brassica incana* flowering top and leaf extracts can induce necrosis in Caco-2 cells. Another study revealed the anticancer effects of ethanolic *Ficus sycomorus* fruit and leaf extracts against HepG2, MCF-7 and Caco-2 cells^30^. Meanwhile, a review highlighted the anticancer effects of white mulberry leaf extract against MCF-7, HepG2 and colorectal cancer cells, HT-29 and HCT-15, through cell cycle arrest, apoptosis and alteration on the proliferation signaling pathway^31^. Interestingly, the *F. sycomorus* and white mulberry leaf extracts also contain components found in BLE including gallic acid, quercetin, rutin and kaempferol^30,31^ while various glucosides of quercetin and kaempferol were found in *B. incana*^29^.

Despite the reported antioxidant activities of BLE, its treatment in Caco-2 cells did not improve the antioxidant status of the cells, but instead reduced it. The reduced antioxidant activity of Caco-2 cells may be associated with the pro-oxidant effects of the treatments. Chikara *et al*.^32^ highlighted that the anticancer activity of phytochemicals can occur through pro-oxidant effects, with the elevation of ROS level. Polyphenolic compounds found in BLE, including GA, quercetin, rutin, protocatechuic acid, and kaempferol have been reported to have pro-oxidant activity^33^. The increase in ROS production and lipid peroxidation observed in this study supported this theory. Although GA IC_50_ had greater effects on the antioxidant activity and ROS production, it did not increase lipid peroxidation as much as BLE IC_50_. We postulate that BLE may be able to induce lipid peroxidation through mechanisms that are not ROS-related.

The antioxidant enzymes tested in this study are involved in the defense against oxidative stress. SOD is responsible for the conversion of superoxide anion into H_2_O_2_ and oxygen while CAT and GPx are involved in detoxifying H_2_O_2_ into water and oxygen^34,35^. The modulation of the activities of these enzymes in Caco-2 cells reflect the concentration of ROS and levels of oxidative stress in the cells. However, the inhibition of SOD was only observed in cells treated with GA implying that BLE may contain compounds including polyphenols that can prevent SOD inhibition. Quercetin and rutin are polyphenolic compounds found in BLE that have been reported to have superoxide anion radical scavenging ability and may be involved in reducing superoxide anion levels^36^. The more drastic alteration of antioxidant enzymes caused by GA suggests that Caco-2 cells treated with GA alone experience higher oxidative stress compared to cells treated with BLE.

Overall, of the 7 genes selected, only the expression of *SLC2A1, AREG* and *ADH4* were consistent with the in-silico analysis previously reported by Kong *et al*.^12^. The previously reported up-regulation of *CXCL8* and *CEACAM1* expression in HepG2 cells^12^ were not observed in any of the treated Caco-2 cells in this study. This could be due to the sensitivity of the microarray analyses used by Kong *et al*.^12^ as well as the differences in cell lines used where HepG2 is an immortalised liver cancer cell while Caco-2 is an epithelial colorectal adenocarcinoma cell. Our group had previously reported that BLE significantly induced the expression of *SLC2A1* gene that encodes the primary glucose transporter, GLUT1, in HepG2 cells^12^. Similar effect was observed in Caco-2 cells treated with BLE. Furthermore, the similar patterns of expression of *SLC2A1* in response to BLE and GA suggests the likelihood of GA being responsible for this effect. However, the elevation of *SLC2A1* expression caused by BLE and GA was not reflected in the glucose concentrations. It is not known at this stage whether the BLE and GA can directly affect the GLUT1 translational process and the transporting activity/efficiency of the mature protein.

Glucose is the substrate of the glycolytic pathway that produces pyruvate as the end-product. Meanwhile, D-lactate is the product of the detoxification of MG through the glyoxalase system^13^. The concentration of MG in Caco-2 cells in this study was not measured as MG is unstable and quickly metabolised once formed^37^. Apart from that, a study on the different MG quantification methods found that each method resulted in different MG values for a given sample^38^. Hence, measuring the concentrations of both intracellular and extracellular glucose, D-lactate and pyruvate, as well as the expression of genes related to glucose transport and glyoxalases may provide indirect information on the effects of BLE and GA on MG metabolism in Caco-2 cells.

Caco-2 cells treated with BLE IC_50_ expressed the greatest increase in expression of *SLC2A1* and exhibited the highest extracellular glucose concentration suggesting the lowest ability of glucose uptake. BLE contains several polyphenolic compounds, such as quercetin and kaempferol, that were reported to have inhibitory effect on glucose transport^39–41^. Meanwhile, the lack of significant changes in glucose concentrations in Caco-2 cells treated with GA is consistent with that reported by Johnston *et al*.^40^. As such, polyphenolic compounds found in BLE, such as GA, may have stimulated the *SLC2A1* gene expression while quercetin and kaempferol inhibited the transport of glucose into Caco-2 cells. The ability of BLE in regulating the expression of *SLC2A1* could be useful to selectively target GLUT 1 in Caco-2 cells.

The expression of genes associated with glyoxalase system was only influenced by GA. Meanwhile, only BLE IC_50_ significantly down-regulated the expression of *ADH4*, a gene associated with MG degradation pathway III. The same gene was reported to be down-regulated in BLE-treated HepG2 cells^12^. Apart from that, the up-regulation of *AKR1B10* expression, another gene involved in MG degradation pathway III, in GA-treated Caco-2 cells was not observed in cells treated with BLE. Together with the significantly induced expression of glyoxalase genes, *GLO1* and *HAGH*, cells treated with GA IC_50_ is likely to be more efficient in detoxifying MG than the other treatments. Hence, GA and BLE may have different effects on MG metabolism. However, despite the alteration of genes involved in MG metabolism, both BLE and GA did not alter the concentrations of intracellular and extracellular D-lactate.

**Figure 7:**
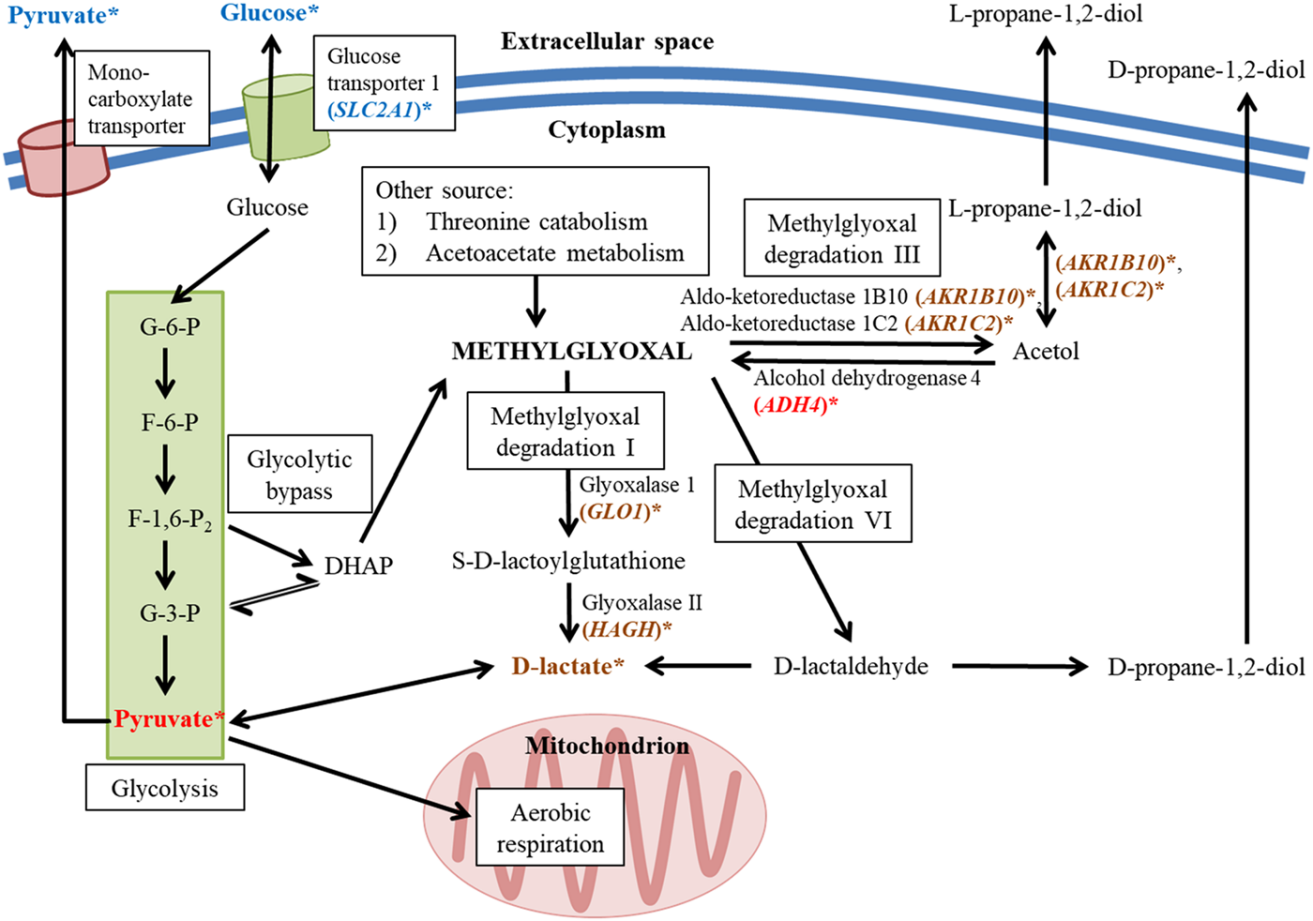
Graphical summary of the effects of BLE on methylglyoxal metabolism. (*) Indicates metabolites or genes that are significantly increased or up-regulated, (*)Indicates metabolites or genes that are significantly decreased or down-regulated and (*) indicates metabolites or genes that are not significantly regulated. The information is derived from CC By 4.0^59^, Schomburg *et al*.^60^, Kong *et al*.^12^, Chakraborty *et al*.^15^ and the current study. DHAP = dihydroxyacetone phosphate.

Cancer cells have higher expression of pyruvate kinase M2 (PKM2) isoform, which is more susceptible to inhibition by proliferation signals, than the M1 (PKM1) isoform. The inhibition of PKM2 leads to lower pyruvate production in favor of cellular growth by channeling glycolytic intermediates toward growth-related biosynthesis^42^. Cytosolic pyruvate can undergo several fates. They can be secreted into the extracellular space via monocarboxylate transporter (MCT), transported into mitochondria via mitochondrial pyruvate carrier (MPC) or undergo enzymatic conversion into lactate, through lactate dehydrogenase (LDH), or alanine, through alanine transaminase, in the cytosol^43,44^. The high concentrations of extracellular pyruvate in the Caco-2 cells treated with BLE IC_50_ and GA IC_50_ suggests that the treatments may have attenuated the production of pyruvate in the cells. Pyruvate production in cancer cells can be elevated through various mechanisms including the inhibition of LDH, inhibition of hypoxia-inducible factor 1 (HIF-1), activation of PKM2 and activation of tumor suppressor p53^45,46^. Apart from that, the shuttling of pyruvate into the extracellular space may be a secretory response towards oxidative stress^47^. In the present study, the exact mechanism involved in the increased production of pyruvate remains unclear. The effects of BLE on MG metabolism, including the changes in metabolite concentrations and gene expression, are summarized in Fig. 7.

All treatment in this study resulted in the alteration of the cell cycle of Caco-2 cells. The effect of GA on Caco-2 cells in this study was similar to that reported by Salucci, Stivala, Maiani, Bugianesi and Vannini^48^ with the exception of the S phase which was increased. The effect of GA on the cell cycle seems to be reversible as the cell cycle distribution of cells treated with GA seems to progress towards that of the untreated control after the treated cells were incubated in fresh media for another 48 hours. To the best of our knowledge, this is the first time that the alteration of Caco-2 cell cycle by BLE is reported. Apart from GA, the pure form of other polyphenolic compounds identified in BLE have been reported to cause cell cycle alteration or arrest in cancer cells. Quercetin has been reported to induce cell cycle arrest in the S phase and decrease in percentage of cells in the G0/G1 phase when treated on colorectal cancer cells, LoVo^49^. Kaempferol was reported to induce cell cycle arrest at the G1 and G2/M phase of colorectal cancer cells, HT-29^50^. Meanwhile, rutin was also reported to induce G2/M arrest in human neuroblastoma cells, LAN-5^51^. Unlike GA, the effects of BLE, especially at IC_50_ concentration, on Caco-2 cell cycle appears to be irreversible with the change of media as the cell cycle distribution of cells treated with BLE at both concentrations became more significantly different than that of the untreated control following the change of media. Taken into consideration of the different effects of each individual pure polyphenolic compound on cell cycle, it is likely that when these compounds are present as a mixture in the BLE used, a synergistic effect occurred.

All treatments resulted in increased percentage of cells undergoing apoptosis with greater apoptotic effects for treatment at higher concentrations. While the apoptotic effect of quercetin-3-O-rutinoside isolated from the fruits of *B. racemosa* had been reported by^8^, this is the first time the apoptotic effect of BLE is reported. GA is known to induce apoptotic effect in Caco-2 cells through caspase-3 activity^21^. Other than GA, pro-apoptotic polyphenolic compounds found in BLE includes rutin, kaempferol, quercetin and ellagic acid. Kaempferol, ellagic acid and quercetin have also been reported to induce apoptotic effect through caspase-3 activation in colorectal cancer cells, HCT116, and leukemia cells, MOLT-4^52,53^. Meanwhile, Chen *et al*.^54^ demonstrated the dose-dependent apoptotic effect of rutin on LAN-5 cells. As such, the apoptotic effect of BLE is likely the synergistic effect of these polyphenolic compounds. However, the apoptotic effect of BLE at IC_50_ may be hindered by the over-expression of AREG gene that encodes amphiregulin. Apart from being an oncogenic factor that promotes tumor development, amphiregulin is also a natural survivor protein that mediates anti-apoptotic signals^55^. Hence, cells treated with BLE and GA at IC_50_ may be stimulated to produce more amphiregulin as a defense mechanism against the apoptotic effects of the treatments.

Yuan *et al*.^56^ described that the synergism in a mixture of compounds may be responsible in enhancing the effectiveness of the mixture as a cure and reducing the toxicity of the individual pure compounds. The authors also highlighted that the synergistic effects could target multiple levels of different pathways and emphasised on the synergy among pure compounds as a method of new drug discovery. More interestingly, a recent study by Addis *et al*.^57^ demonstrated the synergistic effect of waste extract from different medicinal plants in improving fibroblast proliferation and migration which otherwise had been reported to have inhibitory effect on cancer cell lines when used individually. Hence, the different effects of GA and BLE on Caco-2 cells may be due to the synergistic effect of the different compounds, including GA, found in BLE.

It is important for natural compounds to show selective toxicity towards cancer cells before they could be evaluated for therapeutic purposes^58^. The present study was performed with only Caco-2 cells as the sole *in-vitro* model. Additional data on the effects of BLE against other types of colon cancer cells as well as non-cancerous colon cells could further add to our understanding on the anti-proliferative action of BLE against colon cancer. Apart from that, investigation on the sensitivities of other cancer and non-cancerous cell types against BLE could contribute to the development of targeted therapy using BLE. The gene expression analysis in this study encompassed only a targeted, small fraction of genes in Caco-2 cells affected by BLE. The expression of other genes, particularly those involved in cancer cell survival and proliferation, that may have contributed to the anti-cancer effects of BLE could be analysed.

## Conclusion

In the present study, we demonstrated that BLE inhibits the growth of Caco-2 cells. Apart from the anti-proliferative effect, BLE could alter the redox status, modify the glycolytic pathway at biochemical and molecular levels, alter the cell cycle and induce apoptosis in Caco-2 cells. The alteration of the expression of *SLC2A1*, *AREG* and *ADH4* genes were found to be consistent with our previous *in-silico* analysis using HepG2 cells. GA, being the most abundant polyphenolic compound in BLE, shares similar effects with BLE in regulating the expression of *SLC2A1* and *AREG*. However, the two treatments have different effects on the antioxidant enzymes, glucose uptake, alteration of cell cycle, induction of apoptosis and expression of MG degradation genes in Caco-2 cells. This may be the result of synergistic effects of polyphenolic compounds, including GA, or other compounds yet to be identified, present in BLE. These findings may serve as the basis for further investigations to unveil the mode of action of the anti-proliferative effects of BLE. With further chemical and clinical studies, BLE may prove to be a novel chemopreventive agent against colorectal cancer.

## Supplementary information

Supplementary Information.

Supplementary Information 2.
