# Supplementary material for: Evaluation of Anti-proliferative Effects of Barringtonia racemosa and Gallic Acid on Caco-2 Cells
Source: Sci Rep. 2020 Jun 19;10:9987. doi: 10.1038/s41598-020-66913-x (PMC7305318; doi:10.1038/s41598-020-66913-x)
Supplement: Supplementary file 1 — Supplementary Information. [file 41598_2020_66913_MOESM1_ESM.pdf]

Evaluation of Anti-proliferative Effects of *Barringtonia racemosa* and Gallic Acid on Caco-2 Cells

Ivan Y.M. Ho[1], Azlina Abdul Aziz[1] and Sarni Mat Junit[1]*

[1]Department of Molecular Medicine, Faculty of Medicine, University of Malaya, 50603 Kuala Lumpur

*Corresponding author:

Sarni Mat Junit

Email: sarni@um.edu.my,

Tel: +603-79674906; Fax: +603-79674957.

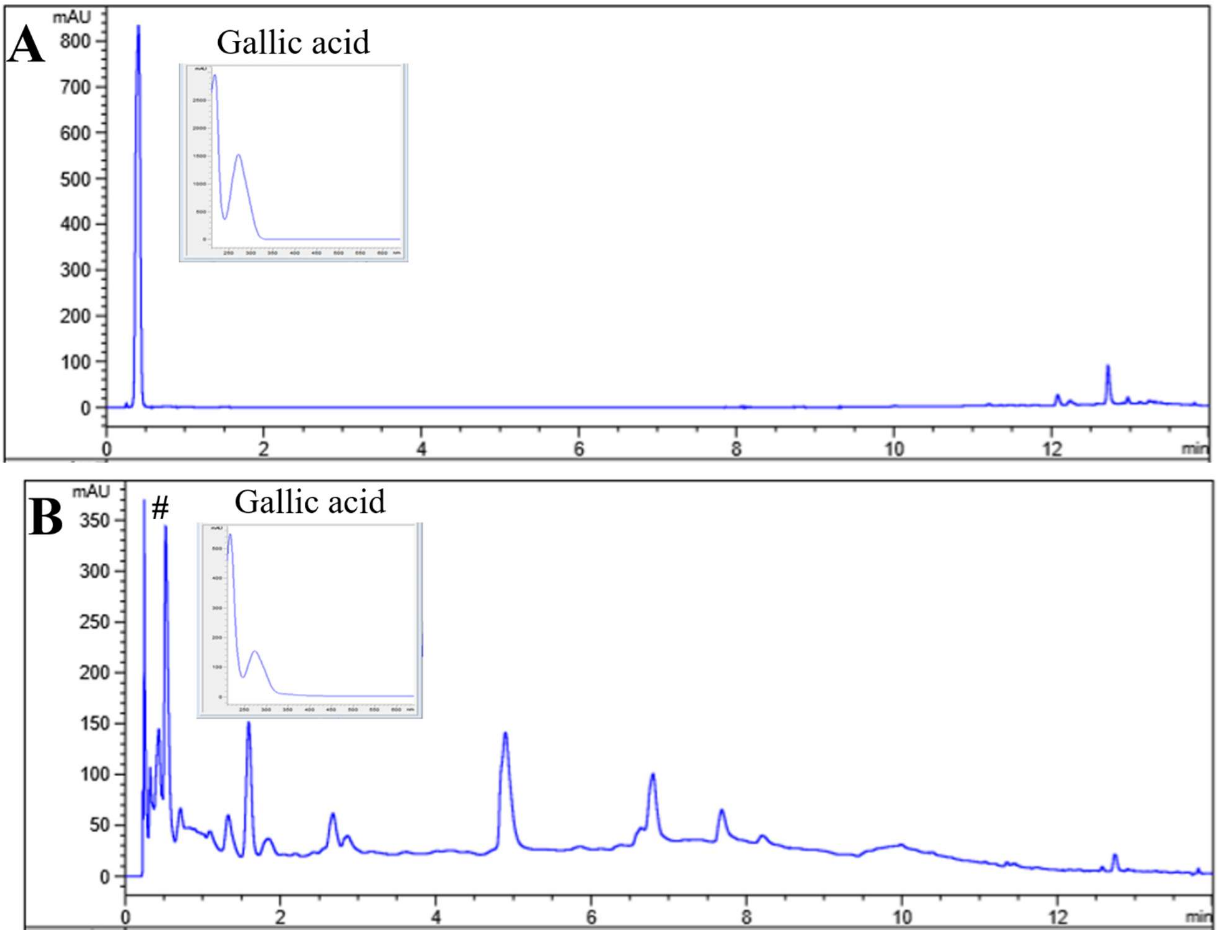

Supplementary Fig. 1 UHPLC analyses of pure polyphenol gallic acid and hydrolyzed BLE. (A) UHPLC chromatogram and UV-vis spectra of pure gallic acid. (B) UHPLC chromatogram and UV-vis spectra of hydrolyzed BLE. (#) indicates gallic acid

UHPLC analysis was performed according to Kong et al. (2012). Five micrograms of BLE was solubilized in 2 ml of 1.2 N HCl containing 20 mM sodium diethyl dithiocarbamate (DETC). The mixture was hydrolyzed at 90 °C for 2 hours in a heating module (Pierce Reacti-Term Heating/Stirring Module No. 1871, Illinois, USA). The hydrolysate was left to cool and filtered through a 0.20 µm polytetrafluoroethylene (PTFE) membrane filter. Separation and analyses were performed using UHPLC (Agilent 1290 Infinity LC system, Agilent Technologies, Germany) fitted with a Zorbax Eclipse Plus C18 column (Agilent Technologies, Germany). Mobile phase A consisted of 0.1% trifluoroacetic acid (TFA) and mobile phase B consisted of 100% acetonitrile. The flow rate was set at 0.6 ml/min and 5 µl of sample was injected into the system. The polyphenolic compounds were separated using a liner gradient system: 5-15% B in 6 min; 15-25% B in 3 min; 25-60% B in 3 min; 60-80% B in 0.6 min; 80-100% in 0.8 min. The detection wavelength was set at 254 nm. Pure gallic acid was prepared in 50% methanol containing 20 mM DETC.
