# Supplementary material for: Evaluation of Anti-proliferative Effects of Barringtonia racemosa and Gallic Acid on Caco-2 Cells
Source: Sci Rep. 2020 Jun 19;10:9987. doi: 10.1038/s41598-020-66913-x (PMC7305318; doi:10.1038/s41598-020-66913-x)
Supplement: Supplementary file 2 — Supplementary Information 2. [file 41598_2020_66913_MOESM2_ESM.pdf]

Evaluation of Anti-proliferative Effects of *Barringtonia racemosa* and Gallic Acid on Caco-2 Cells

Ivan Y.M. Ho[1], Azlina Abdul Aziz[1] and Sarni Mat Junit[1]*

[1]Department of Molecular Medicine, Faculty of Medicine, University of Malaya, 50603 Kuala Lumpur

*Corresponding author:

Sarni Mat Junit

Email: sarni@um.edu.my,

Tel: +603-79674906; Fax: +603-79674957.

Supplementary Table 1. List of genes used in qRT-PCR, the primer sequence, corresponding protein and protein functions of each gene. *GAPDH* and *RPLP0* are housekeeping genes used for normalisation of gene expressions.

No.	Gene	Primer Sequence	Corresponding Protein
1	*GAPDH*	Forward:<br>5'-TGC ACC ACC AAC TGC TTA GC-3'<br>Reverse:<br>5'-GGC ATG GAC TGT GGT CAT GAG-3'	Glyceraldehyde-3- phosphate dehydrogenase
2	*RPLP0*	Forward:<br>5'-GCA ATG TTG CCA GTG TCT G-3'<br>Reverse:<br>5'-GCC TTG ACC TTT TCA GCA A-3'	60S acidic ribosomal protein P0
3	*AKR1B10*	Forward:<br>5'-CCA GGT TCT GAT CCG TTT CC-3'<br>Reverse:<br>5'-ACA ATG CGT GCT GGT GTC A-3'	Aldo-keto reductase family 1 member B10
4	*AKR1C2*	Forward:<br>5'-GCC GTC AAA TTG GCA ATA GAA G-3'<br>Reverse:<br>5'-AAC CTG CTC CTC ATT ATT GTA AAC-3'	Aldo-keto reductase family 1 member C2
5	*ADH4*	Forward:<br>5'-GAA ACC ATG AAA GCA GCC CT-3'<br>Reverse:<br>5'-CCA ACC ACC AAA GAA TGT TCC-3'	Class II alcohol dehydrogenase 4 pi-subunit
6	*GLO1*	Forward :<br>5'-CCG CCA TGA TTC ACA TTT GA-3'<br>Reverse :<br>5'-GTT GGC ATG GCC TTT CCA-3'	Glyoxalase I
7	*HAGH*	Forward :<br>5'-CTG CCC TGA CCG ACA ACT AC-3'<br>Reverse :<br>5'-GTT TCA CCC CGT GCT TTC TC-3'	Glyoxalase II
8	*SLC5A1*	Forward:<br>5'-CTG GCA GGC CGA AGT ATG-3'<br>Reverse:<br>5'-CCA CTT CCA ATG TTA CTA GCA AAG-3'	Sodium-dependent glucose transporter 1 (SGLT1)
9	*SLC2A1*	Forward:<br>5'-GGT TGT GCC ATA CTC ATG ACC-3<br>Reverse:<br>5'-CAG ATA GGA CAT CCA GGG TAG C-3'	Glucose transporter 1 (GLUT1)
10	*CXCL8*	Forward:<br>5'-CAC CGG AAG GAA CCA TCT CA-3'<br>Reverse:<br>5'-GGA AGG CTG CCA AGA GAG C-3'	Interleukin-8 (IL8)
11	*AREG*	Forward:<br>5'-CGG GAG CCG ACT ATG ACT ACT C-3'<br>Reverse:<br>5'-GGG CTT AAC TAC CTG TTC AAC TCT G-3'	Amphiregulin
12	*CEACAM1*	Forward:<br>5'-AGC AAC TGG ACA GTT CCA TGT ATA-3'<br>Reverse:<br>5'-AGG TAG GTT GTG TCC TGA GTC T-3'	Carcinoembryonic antigen-related cell adhesion molecule 1
